# Influence of cachexia on immunotherapy efficacy and prognosis for malignant tumors of the digestive system

**DOI:** 10.1002/cnr2.2100

**Published:** 2024-05-22

**Authors:** Zhirui Tao, Zhiqin Chen, Yong Gao, Ming Quan

**Affiliations:** ^1^ Department of Oncology Shanghai East Hospital, Tongji University School of Medicine Shanghai China

**Keywords:** cachexia, digestive system, immunotherapy, survival, tumor

## Abstract

**Background:**

The presence of cancer cachexia is a significant adverse prognostic indicator in patients with malignant tumors. Cancer cachexia is a multifactorial syndrome characterized by a constant loss of skeletal muscles with or without a loss of weight, leading to immune dysfunction. We performed a retrospective study to investigate the influence of cachexia on the immunotherapy efficacy and prognosis for malignant tumors of the digestive system.

**Methods:**

The present study adopts a cross‐sectional design. The prognosis data of patients with advanced cancer of the digestive system who received immunotherapy from September 2021 to December 2022 were analyzed. Cachexia was calculated using the change of the area of the psoas major muscle (PMMA) or the weight. We measured the change at the beginning of immunotherapy and at least 2 cycles afterward. The participants were categorized into the cachexia group and control group based on the evaluation criteria. Kaplan–Meier and Log‐rank methods were used for survival analysis. Cox proportional hazard model as a method to assess the contribution of different clinical factors to overall survival (OS) and progression‐free survival (PFS).

**Results:**

A total number of 98 patients, including esophageal carcinoma (4, 4%), gastric (36, 37%), colorectal (51, 52%), and other cancer types (7, 7%), were enrolled. Fifty‐four patients were diagnosed with non‐cancer cachexia, and the cancer cachexia group included 44 patients. The median PFS in the cachexia group was shorter than that in the control group (130 days vs. 212 days). Their difference was not significant (*p* = .321). The survival rate of the patients without cachexia was longer than of those with cachexia (*p* = .027). The level of albumin and the number of metastatic organs were related to PFS (*p* = .020, *p* = .029). The albumin level was significantly associated with the OS of patients (*p* = .003).

**Conclusions:**

The presence of cachexia was significantly associated with poor OS in patients with malignant tumors of the digestive system who received immunotherapy, not with PFS or the response to immunotherapy.

**Abbreviations**
ACT: adoptive cell transfer
AUC: area under the curve
BMI: body mass index
CCS: cancer cachexia syndrome
CEA: carcinoembryonic antigen
CR: complete response
CT: computed tomography
DCR: disease control rate
ECOG: Eastern Cooperative Oncology Group
HB: hemoglobin
ICIs: immune checkpoint inhibitors
LDH: lactate dehydrogenase
MRI: magnetic resonance imaging
NLR: neutrophil‐to‐lymphocyte ratio
ORR: objective response rate
OS: overall survival
PD: progressive disease
PD‐1: programmed death protein 1
PD‐L1: programmed death ligand 1
PFS: progression‐free survival
PMMA: the area of the psoas major muscle
PR: partial response
PS: performance status
PTL: platelet
ROC: receiver operating characteristic curve
SD: stable disease
SD: standard deviation
TNM: tumor‐node‐metastasis
UICC: Union for International Cancer Control
WBC: white blood cell

## 1 INTRODUCTION

The global yearly incidence of cancer has been increasing, and the number of cancer deaths is also on the rise. Among them, digestive system tumors account for more than 20%. It is estimated that at least 30% of cancer deaths each year are due to digestive system tumors.^1^ Surgery, radiotherapy, and chemotherapy have been the three most important choices of treatment for cancer patients. With the advances in the research on tumor pathogenesis, the concept of immunotherapy has been proposed.^2^ Oncolytic virus therapies, cancer vaccines, cytokine therapies, adoptive cell transfer (ACT), and immune checkpoint inhibitors (ICIs) are several major types of immunotherapy applied in cancer treatment. Now, ICIs are entering medical practice and have become one of the most essential immunotherapies.^3^ ICIs suppress the binding between programmed death protein 1 (PD‐1) and programmed death ligand 1 (PD‐L1), thereby restoring an effective antitumor T‐cell response.^4^ Indeed, ICIs have achieved remarkable clinical outcomes,^5^ and antibodies targeting PD‐1 or PD‐L1 have been approved to treat multiple cancers.^6^ A breakthrough in immunotherapy has brought about many benefits for tumor patients.^5^ This rapid immunotherapy development is significant in those with advanced or metastatic tumors, whose prognosis is significantly improved.^7^ Nevertheless, only a small percentage of patients treated with ICIs experience lasting clinical benefits. Currently, the RECIST criteria are used to evaluate the effect of immunotherapy based on the tumor size change before and after treatment.^8^ Notably, some patients receiving immunotherapy observed an increase in the tumor size or the appearance of new lesions. However, tumor regression was observed at the subsequent follow‐up examinations. This phenomenon has been described as “pseudo‐progression.”^9^ Nonetheless, the existing criteria for evaluating immunotherapy outcomes have limitations. Other clinical indicators to predict survival after immunotherapy are lacking. To date, few studies have been conducted on predicting the effect of immunotherapy based on the general clinical manifestations of patients, and even fewer investigations have been performed on gastrointestinal malignancies.

Abundant evidence has shown that muscle and weight loss can affect immunotherapy outcomes.^10^, ^11^ Malnutrition, especially cachexia, may negatively influence the efficacy of immunotherapy by affecting the immune function of patients. However, the exact mechanism of action is unclear.^12^ Cachexia is a syndrome of continuous weight loss and muscle wasting. It is a manifestation of irreversible malnutrition that cannot be improved by regular nutritional support.^13^ Cachexia has a high incidence of malignancies and is associated with significant adverse survival outcomes. At least half of patients with advanced malignancies develop cachexia, and 20% die from it.^14^

Loss of skeletal muscle is considered an effective indicator of disease status in cancer patients.^15^ However, objectively assessing the progress of skeletal muscle loss is not easy. Recent studies have shown a significant correlation between the third lumbar (L3) monolithic skeletal muscle area and systemic skeletal muscle areas. As an indicator of muscle loss, the area of the psoas major muscle (PMMA) can substitute for whole skeletal muscle mass.^16^, ^17^ Moreover, computed tomography (CT) is a routine examination used to evaluate the therapeutic effect of patients, which is easy to perform and has important practical significance.

Up to now, no study has investigated weight loss combined with PMMA changes to evaluate the occurrence of cachexia in patients with digestive system tumors. The purpose of this study was to evaluate cachexia and examine the impact of cachexia on the prognosis of patients with malignant tumors of the digestive receiving immunotherapy.

## 2 METHODS

### 2.1 Patient selection

The study was approved by the Ethics Committee of Shanghai East Hospital (Grant No. 2024‐035). We retrospectively accrued consecutive patients with malignant tumors of the digestive system, including colorectal, gastric, esophageal, pancreatic, liver, and gallbladder cancer, who received PD‐1/PD‐L1 inhibitors either as monotherapy or in combination with chemotherapy at the Shanghai East Hospital between September 2021 and December 2022. The study included all patients who underwent initial immunotherapy treatment at our hospital. Patients who were undergoing hormone therapy or had incomplete medical information were excluded from the study. Additionally, patients with metabolic disorders such as hyperthyroidism and adrenal insufficiency were also excluded. Covariates and potential factors were selected based on the results of previously published studies. The baseline data from the first assessment were analyzed, including patient characteristics (age, sex, Eastern Cooperative Oncology Group [ECOG] performance status [PS], and body mass index [BMI]), disease characteristics (number of metastatic organs, tumor‐node‐metastasis [TNM] classification as proposed by the 8th edition of Union for International Cancer Control [UICC], type of tumor), treatment characteristics (progression‐free survival [PFS], disease control rate [DCR], objective response rate [ORR], overall survival [OS]). The level of lactate dehydrogenase (LDH), total protein, albumin, neutrophil, lymphocyte, neutrophil‐to‐lymphocyte ratio (NLR), hemoglobin (HB), white blood cell (WBC), platelet (PLT), and carcinoembryonic antigen (CEA) were also measured in routine examinations performed before immunotherapy. Patients were classified in a dichotomous fashion based on their gender (male vs. female), PS (0–1 vs. ≥2), number of metastatic organs (0–2 vs. >2), baseline BMI values (≤20 vs. >20 kg/m^2^), and baseline D‐dimers values (≤0.5 vs. >0.5). The data were obtained from the electronic medical records in our hospital. Informed consent was obtained from all participants or their legal guardians.

### 2.2 Cachexia assessment

Cancer cachexia is a multifactorial syndrome characterized by skeletal muscle loss with or without a loss of weight, leading to immune dysfunction. According to previously published studies,^18^, ^19^ the definition and classification of cancer cachexia are based on the following criteria: (1) the change in PMMA was used as an assessment criterion for cachexia; or (2) a weight loss >5% over the past 6 months (in the absence of simple starvation); or (3) BMI < 20 and any degree of weight loss >2%.

**FIGURE 1:**
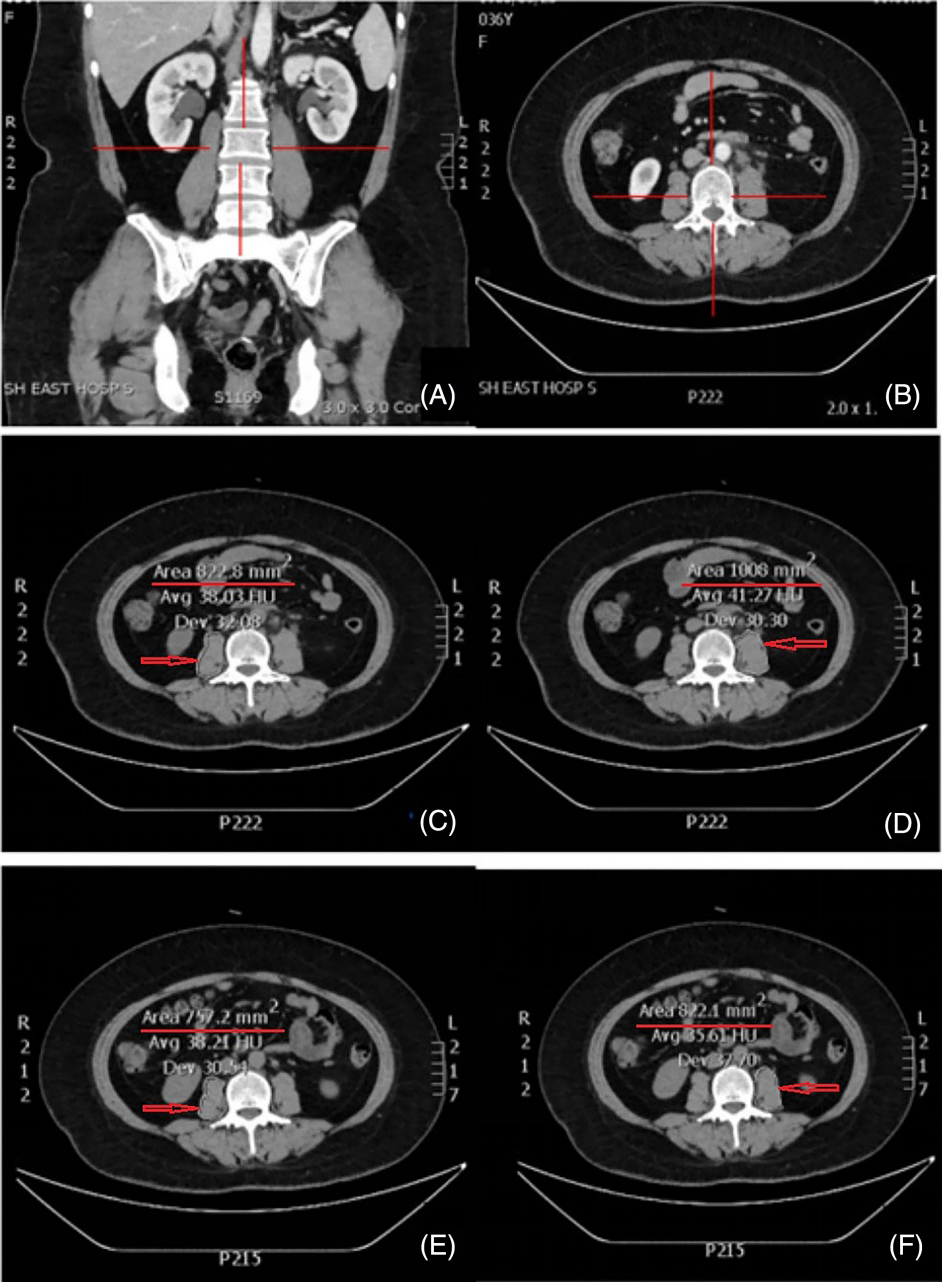
Method for locating the level of the third lumbar spine and the PMMA and PMMA by measuring the skeletal muscle thickness at the level of the upper L3 margin. (A) The red line cross is the coronal position of the third lumbar spine. (B) The red line cross is the horizontal position of the third lumbar spine. (C) 8.228 cm^2^ is the area of the right psoas before treatment. (D) 10.08 cm^2^ is the area of the left psoas before treatment. (E) 7.572 cm^2^ is the area of the right psoas after two treatment cycles. (F) 8.221 cm^2^ is the area of the left psoas after two treatment cycles. In the case presented before treatment, the PMMA was 18.308 cm^2^ (PMMA = 8.228 cm^2^ + 10.08 cm^2^ = 18.308 cm^2^); after two treatment cycles, the PMMA was 15.793 cm^2^ (PMMA = 7.572 cm^2^ + 8.221 cm^2^ = 15.793 cm^2^). The change rate of PMMA was calculated as follows: (1–15.793/18.308) × 100 = 13.737%. PMMA, area of the psoas major muscle.

CT reading software was used to calculate the PMMA at the level of the upper L3 margin (Figure 1). PMMA was evaluated before initial immunotherapy and once after 2 cycles, and cachexia was considered to have occurred if its rate of change ≥10%.

### 2.3 Outcome assessment

Primary endpoints were OS and PFS, while secondary endpoints were ORR and DCR. We defined OS as the time from the start of therapy to death and PFS as the time from the first use of immunotherapy to disease progression or death from any cause. Patients alive were censored at the last known date to be alive. The response to treatment was evaluated according to the RECIST 1.1 criteria. The tumor response was assessed as complete response (CR), partial response (PR), stable disease (SD), and progressive disease (PD) using the patient's CT scan or magnetic resonance imaging (MRI) results. ORR was defined as the sum of CR and PR. DCR was the proportion of patients without disease progression in the total population.

### 2.4 Statistical analysis

Descriptive statistics data are presented as mean ± standard deviation (SD), or in case of skewed distributions, as median (range) or frequencies (percentages). Comparisons between groups were performed using the independent *t*‐test for continuous normally distributed variables or with the Mann–Whitney U‐test. For measurement data, the differences in the patient characteristics between the cachexia and non‐cachexia groups were analyzed using the χ^2^ test or the Fisher exact test. Spearman correlation analysis was conducted to test the associations between other factors, the data of which were collected, and BMI. Additionally, we evaluated the association between other factors and PMMA. The probabilities of OS and PFS were calculated using the Kaplan–Meier method. The log‐rank test was applied to test the survival curves of patients with or without cachexia. Age, BMI, PMMA, number of metastatic organs, PS, and albumin were included as covariates in the univariate analysis (Cox proportional hazard model) to assess the contribution of different clinical factors to OS and PFS. The Cox proportional hazard model was also performed by including potential contributing factors in the multivariate analysis, identified as factors yielding a *p*‐value <.10 in the univariate analysis. The diagnostic accuracy of weight loss combined with PMMA changes to evaluate the occurrence of cachexia was determined by obtaining the largest possible area under the curve (AUC) in the receiver operating characteristic curve (ROC) analysis. AUC values ≥0·90 were considered excellent， ≥ 0·70 were considered good, ≥0·50 were considered fair, and <0·50 were considered poor. All analyses were performed with SPSS statistical software (IBM version 26.0 for Windows). *p*‐values <.05 were considered to indicate statistically significant differences.

## 3 RESULTS

### 3.1 Patient characteristics

**FIGURE 2:**
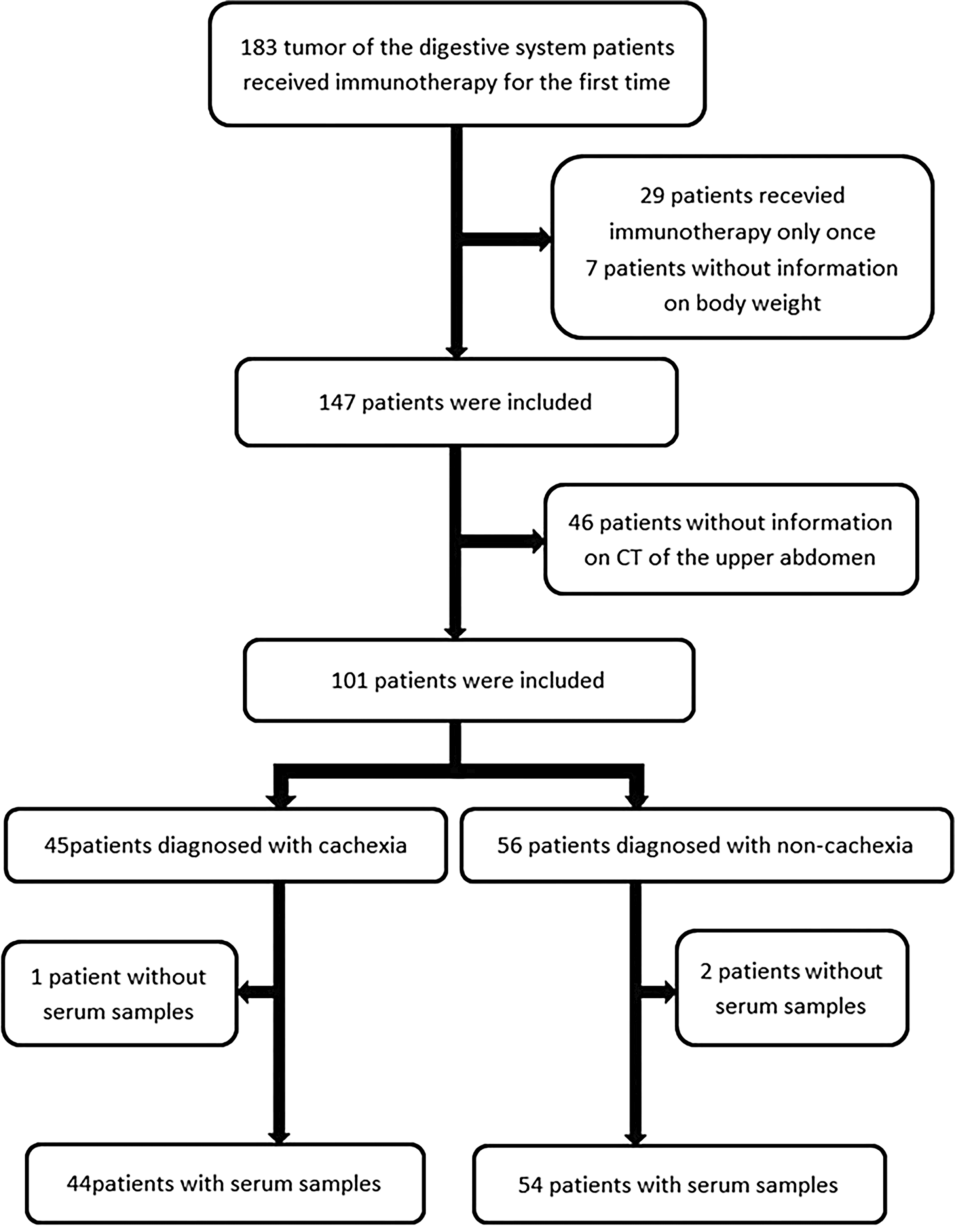
Flow diagram of the study.

In our study, we included data from 183 patients with digestive system tumors who received immunotherapy for the first time between September 2021 and December 2022. Of these 183 patients, 82 were excluded, and only one immunotherapy cycle was recorded for 29 patients; no weight information was available for 7 patients, and 46 patients were without information on CT of the upper abdomen. Finally, 101 patients were included. However, three patients were without analyzed serum samples. Finally, 98 patients were enrolled in this investigation (Figure 2), including esophageal carcinoma (4, 4%), gastric (36, 37%), and colorectal (51, 52%) and other cancer type (7, 7%). Fifty‐four patients were diagnosed with non‐cancer cachexia, and the cancer cachexia group included 44 patients (45%). The cachexia group consisted of 42 patients at clinical stage IV and 2 patients at stage III. In contrast, the non‐cachexia group included 52 patients at stage IV and 2 patients at stage III. All patients were followed up until April 8, 2023. The follow‐up duration ranged from 46 to 576 days. The median follow‐up period was 262.5 days. Of the 98 patients in the whole group, 26 were lost to follow‐up, with a loss rate of 27%. Furthermore, 17 patients died, 12 of whom were in the cachexia group. The data were taken before the death of the patients.

The baseline characteristics of the 98 patients are presented in Table 1. The mean age at the start of treatment was 62 ± 13 years, and 63% were male; 60% of the patients had a PS of 0–1. More than 50% of the patients in the non‐cachexia group were diagnosed with colorectal cancer. The number of colorectal cancer patients in the cachexia group also was the highest (Figure S1).

**TABLE 1:** Baseline characteristics of the patients who received immunotherapy.

	All patients	With cachexia	Without cachexia	*p*‐value
Gender				0.529
Male, *N* (%)	62 (63%)	26 (59%)	36 (67%)	
Female, *N* (%)	36 (37%)	18 (41%)	18 (33%)	
Age(years), median(range)	65 (28–91)	65 (28–91)	64 (36–88)	0.492
PS, *N* (%)				0.678
0–1	59 (60%)	25 (57%)	34 (63%)	
≥2	39 (40%)	19 (43%)	20 (37%)	
BMI (kg/m^2^), mean ± SD	21.91 ± 3.48	21.82 ± 3.24	21.99 ± 3.24	0.622
BMI (kg/m^2^), *N* (%)				0.517
>20	68 (69%)	29 (66%)	39 (72%)	
≤20	30 (31%)	15 (34%)	15 (28%)	
PMMA (cm^2^), mean ± SD	12.73 ± 4.29	12.50 ± 4.62	12.93 ± 4.03	0.623
Metastatic organs, *N* (%)				0.916
0–2	74 (76%)	33 (75%)	41 (76%)	
>2	24 (24%)	11 (25%)	13 (24%)	
LDH (U/L), Median (range)	205 (76.00–2990.00)	205.50 (76.00–1453.00)	211.50 (103.00–2990.00)	0.972
Total protein (g/L), mean ± SD	68.55 ± 8.69	69.55 ± 9.32	67.74 ± 8.14	0.306
Albumin(g/L), median (range)	39.40 (25.00–48.50)	39.00 (28.00–48.30)	39.50 (25.00–48.50)	0.568
Neutrophil (10^^^9/L), median k(range)	4.91 (1.36–35.70)	5.00 (1.36–21.30)	4.59 (1.68–35.70)	0.551
Lymphocyte (10^^^9/L), median (range)	1.09 (0.21–6.79)	1.04 (0.41–6.79)	1.16 (0.21–3.98)	0.537
NLR (ratio), median (range)	4.31 (0.77–28.86)	4.85 (0.77–24.86)	4.04 (0.90–28.86)	0.440
HB (g/L), mean ± SD	110.77 ± 21.39	111.14 ± 20.30	110.46 ± 22.42	0.878
WBC (10^^^9/L), median (range)	6.39 (2.20–29.31)	6.58 (3.12–19.58)	6.04 (2.20–29.31)	0.255
PLT (10^^^9/L), median (range)	196.00 (59.00–565.00)	201.50 (71.00–565.00	193.00 (59.00–502.00)	0.447
CEA (ng/mL), Median (range)	9.01 (0.80–22796.00)	6.55 (0.90–22796.00)	9.35 (0.80–7455.00)	0.565
D‐dimers				0.256
≤0.5	26 (27%)	9 (20%)	17 (31%)	
>0.5	72 (73%)	35 (80%)	37 (69%)	

Abbreviations: BMI, body mass index; CEA, carcinoembryonic antigen; HB, hemoglobin; LDH, lactate dehydrogenase; NLR, Neutrophil‐to‐lymphocyte ratio; PLT, platelet; PMMA, the area of the psoas major muscle; PS, performance status; WBC, white blood cell.

### 3.2 ROC analysis

**FIGURE 3:**
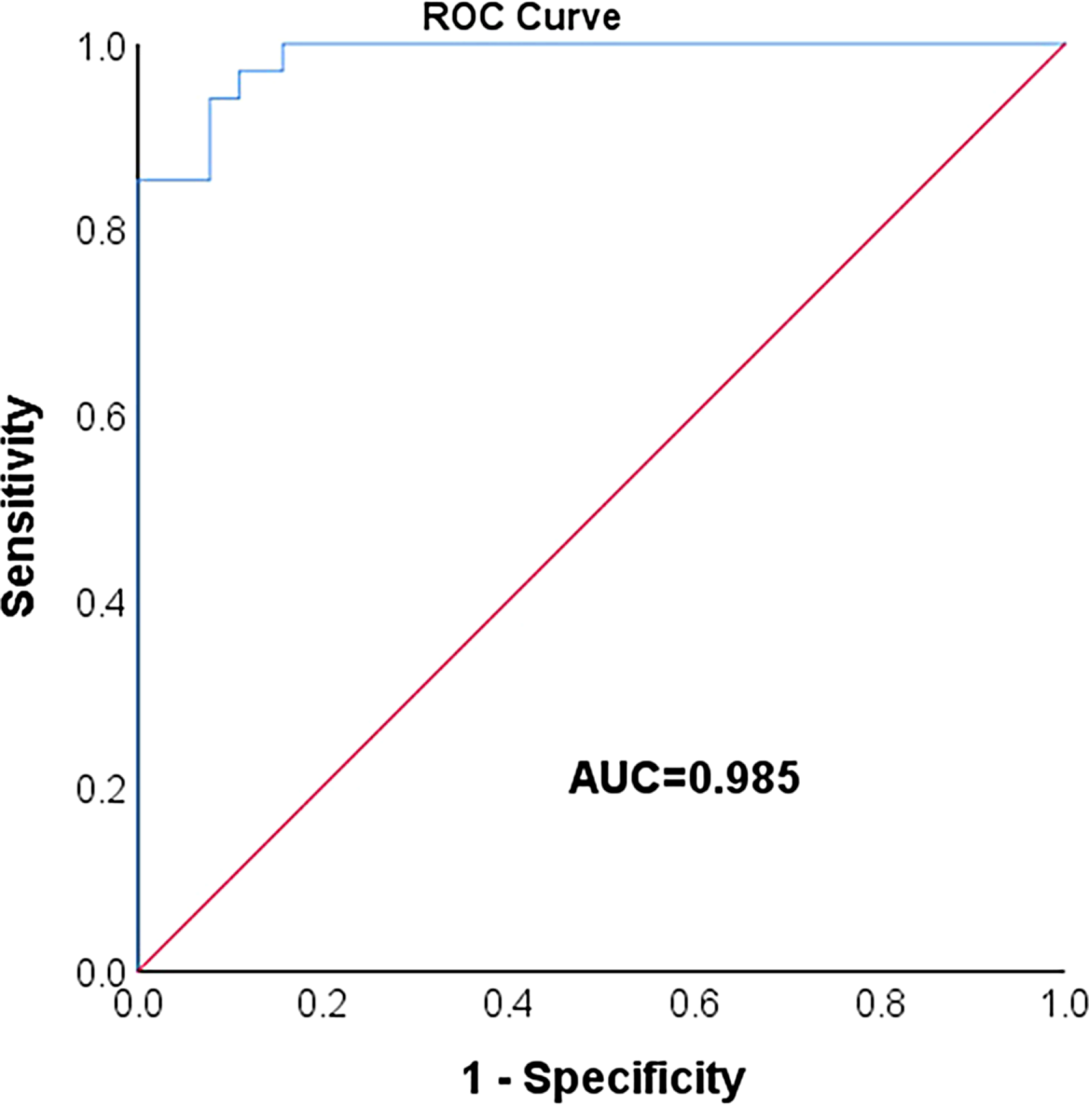
ROC curve of the criteria for cachexia. The area under the ROC curve was utilized to judge the advantages and disadvantages of the criteria. ROC, receiver operating curve.ROC, receiver operating characteristic.

ROC analysis was performed to explore the potential predictive role of our method for the discrimination between patients with or without cachexia. At the optimal cut‐off values for body weight combined with PMMA could significantly and easily distinguish between patients with or without cachexia (AUC = 0.985, 95% confidence interval [CI] = 0.969–1, *p* < .001), with a sensitivity of 85.3% and a specificity of 98.4% (Figure 3).

### 3.3 Effect of cachexia on the response and survival outcomes

**FIGURE 4:**
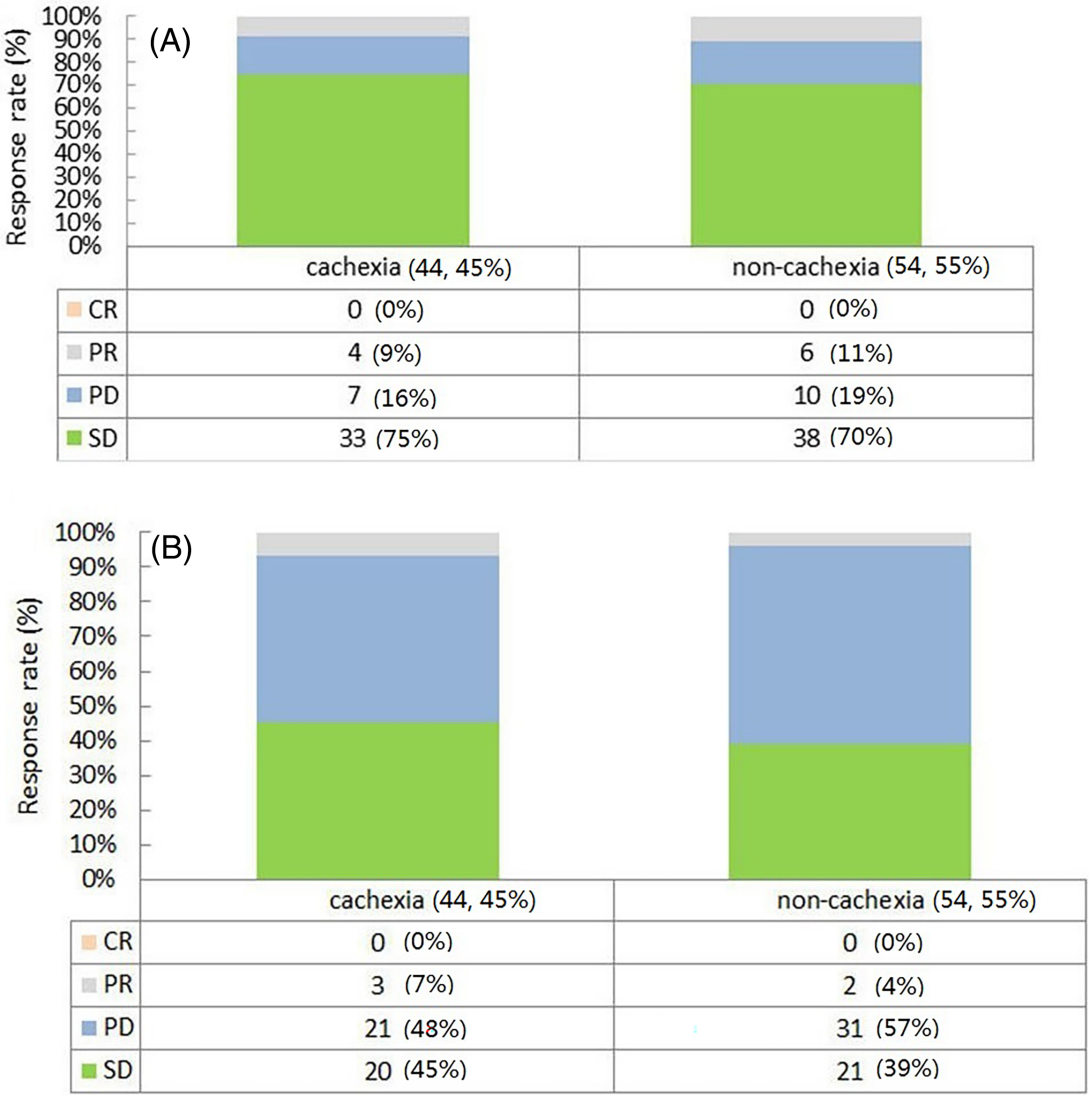
Distribution of responses after two cyclical immunotherapy (A) and distribution of responses at last follow‐up (B). CR, complete response; PD, progressive disease; PR, partial response; SD, stable disease.

The first assessment of response was performed after two treatment cycles. The response assessment of patients in the cachexia group at the initial evaluation is depicted in Figure 4A. The overall distribution of response rate did not exhibit a significant difference between the cachexia and non‐cachexia groups (*p* = .846). The final evaluation of the response was made at the last follow‐up. The response assessment of patients in the cachexia group at the last evaluation is depicted in Figure 4B. We found no difference between the overall distribution of the response rate of the two groups (*p* = .628). DCR was 52% in the cachexia group and 43% in the non‐cachexia group; the ORR values were 7% and 4%, respectively. Neither ORR nor DCR differed between the two groups (ORR: *p* = .654; DCR: *p* = .417).

**FIGURE 5:**
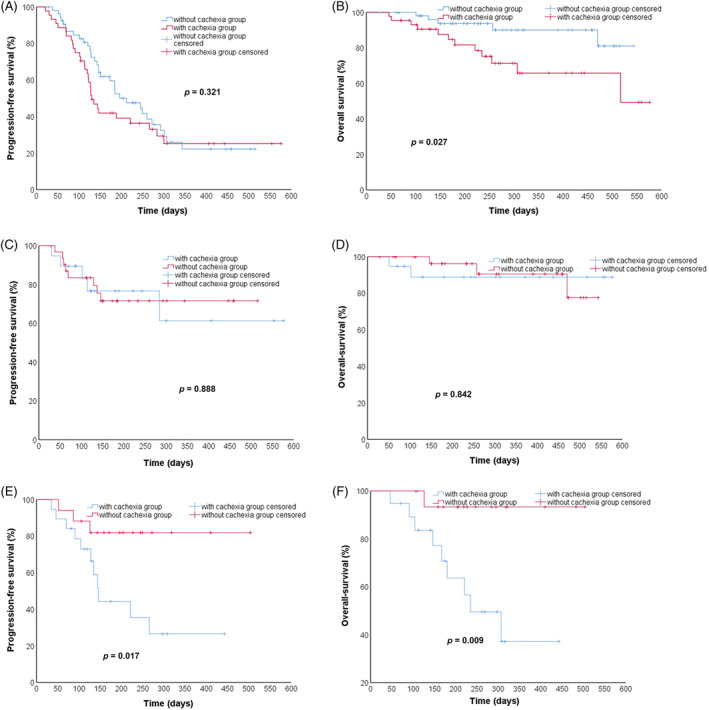
Kaplan–Meier estimates survival curves for all patients (*n* = 98) PFS (A) and OS (B); colorectal cancer (*n* = 51) PFS (C) and OS (D); gastric cancer (*n* = 36) PFS (E) and OS (F). OS, overall survival; PFS, progression‐free survival.

**FIGURE 6:**
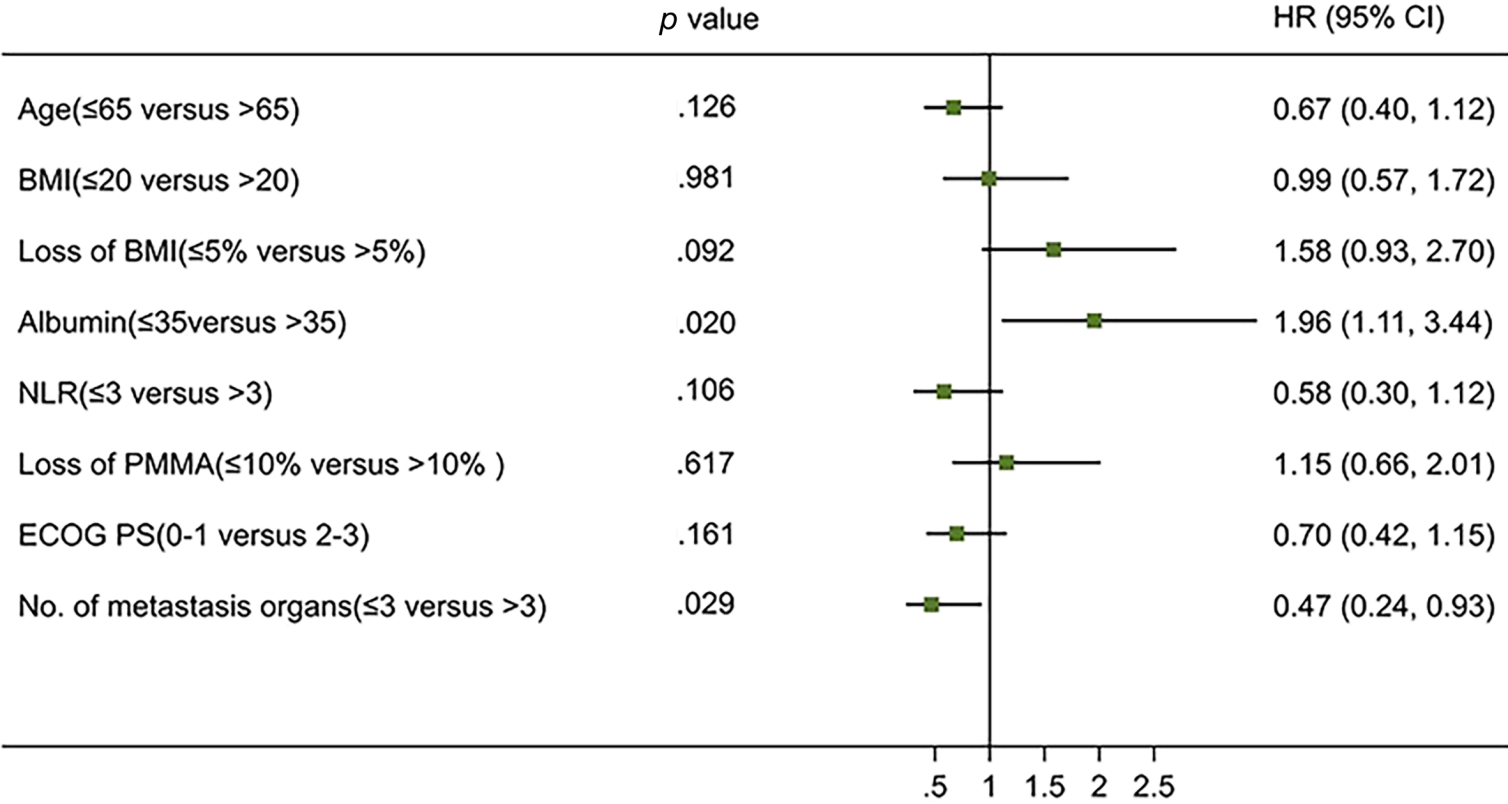
Univariate analyses of the factors associated with progression‐free survival (PFS) (*N* = 98).

**FIGURE 7:**
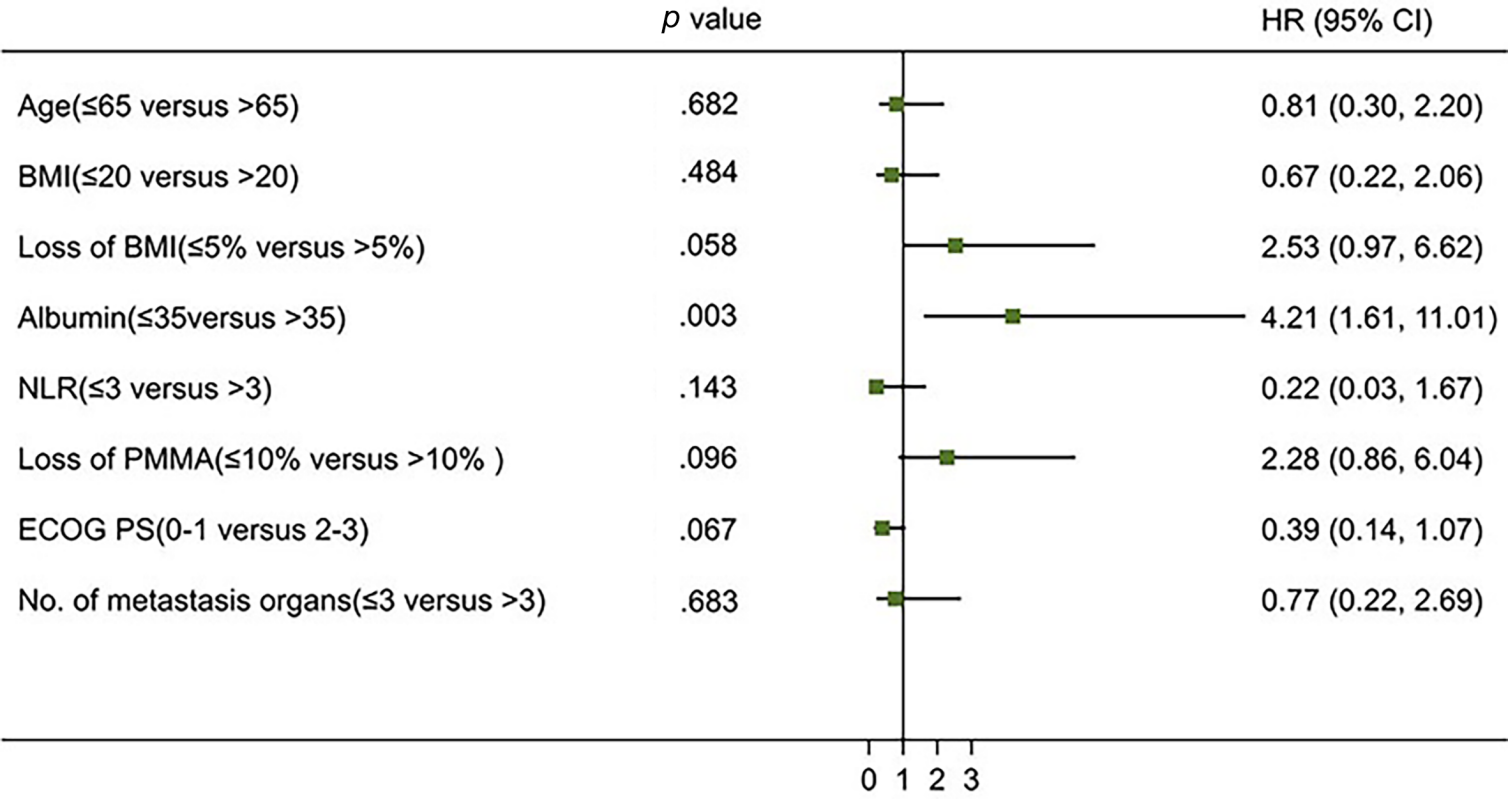
Univariate analyses of the factors associated with overall survival (*N* = 98).

The median PFS in the non‐cachexia group was longer than that of the cachexia group (212 days vs. 130 days). However, their difference was not statistically significant (*p* = .321). In the non‐cachexia group, the 6‐month survival rate was 93.6%, and the 1‐year survival rate was 90.0%. The six‐month and one‐year survival rates in the cachexia group were 81.6% and 65.8%, respectively. The survival rate of the patients without cachexia was longer than that of those with cachexia (*p* = .027) (Figure 5B). Univariate Cox regression analysis revealed that the level of albumin (*p* = .020) and the number of metastatic organs (*p* = .029) were related to PFS (Figure 6). The level of albumin was also associated with OS (*p* = .003) (Figure 7). The multivariate analysis of all these potential factors identified only the level of albumin during treatment as a strong independent negative predictor for OS (*p* = .003). The level of albumin and the number of metastatic organs were independent influencing factors of PFS (Table 2).

**TABLE 2:** Multivariate analyses of factors associated with PFS and OS (*N* = 98).

		PFS		OS	
Variables		HR [95% CI]	*p*‐value	HR [95% CI]	*p*‐value
Albumin, g/L	≤35 versus >35	1.929 [1.094–3.403]	.023	4.209 [1.609–11.01]	.003
No. of metastasis organs	≤3 versus >3	0.478 [0.241–0.948]	.035		

Abbreviations: OS, overall survival; PFS, progression‐free survival.

A subgroup analysis for gastric cancer patients and colorectal cancer patients who received ICIs for survival curves. Results show that for gastric cancer, both PFS and OS were shorter in patients with cachexia: PFS 219 days, 95% CI 144–295 versus 429 days, 95% CI 352–506, *p* = .017 (Figure 5E); OS 280 days, 95% CI 210–352 versus 479 days, 95% CI 431–527, *p* = .009 (Figure 5F). Conversely, no differences were found between cachexia and non‐cachexia patients both for PFS and OS in colorectal cancer patients who underwent ICIs: PFS 415 days, 95% CI 297–533 versus 394 days, 95% CI 323–466, *p* = .888 (Figure 5C); OS 520 days, 95% CI 447–593 versus 502 days, 95% CI 458–546, *p* = .842 (Figure 5D).

### 3.4 Correlations of albumin and NLR levels with cachexia

**FIGURE 8:**
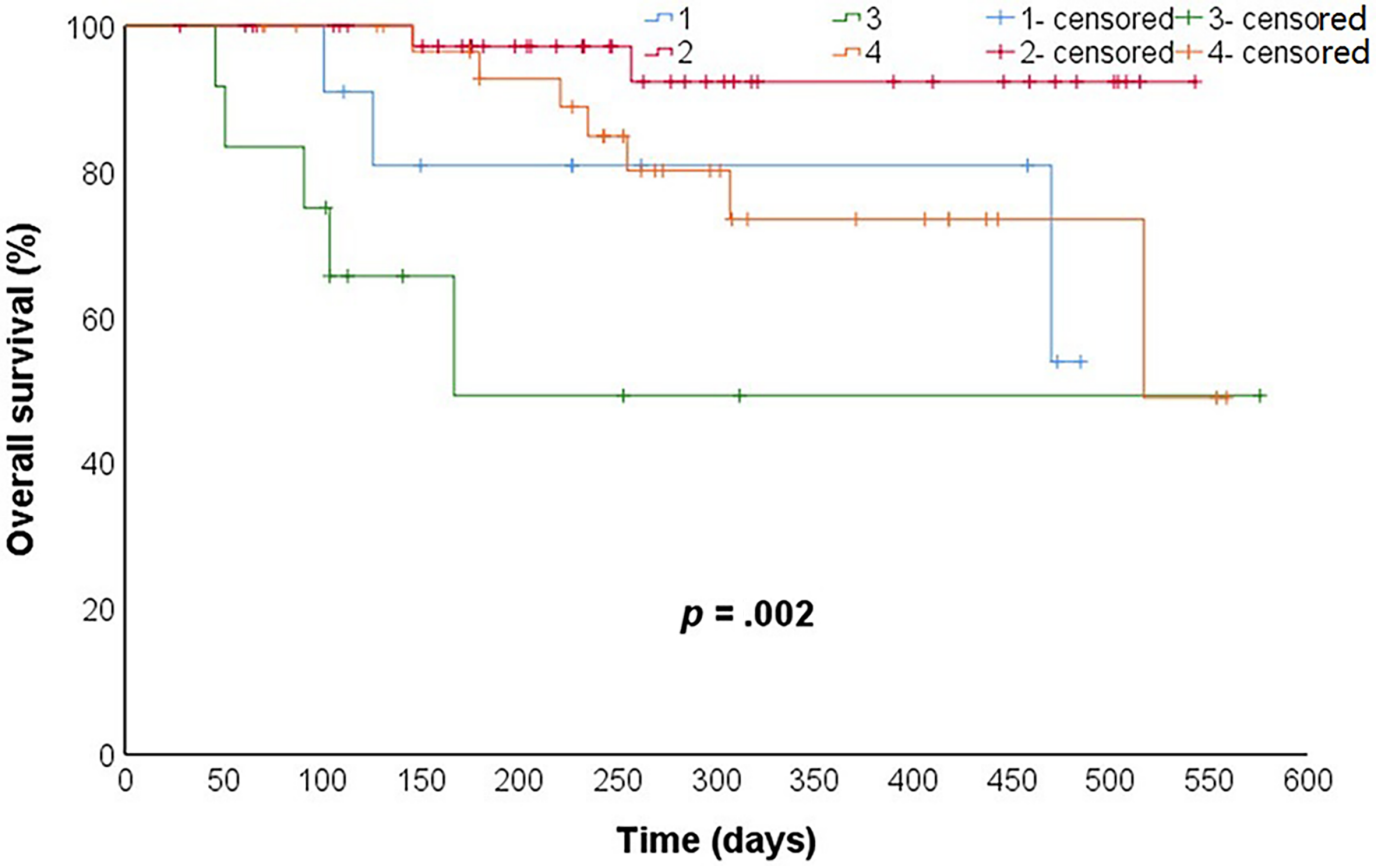
Kaplan–Meier estimates of overall survival in the following groups: 1: without cachexia and albumin ≤35 g/L; 2: without cachexia and albumin >35 g/L; 3: with cachexia and albumin ≤35 g/L; 4: with cachexia and albumin >35 g/L. (a) 1 contrast with 2, *p* = .042; (b) 1 contrast with 3, *p* = .234; (c) 1 contrast with 4, *p* = .592; (d) 2 contrast with 3, *p* < .001; (e) 2 contrast with 4, *p* = .090; and (f) 3 contrast with 4, *p* = .022.

**FIGURE 9:**
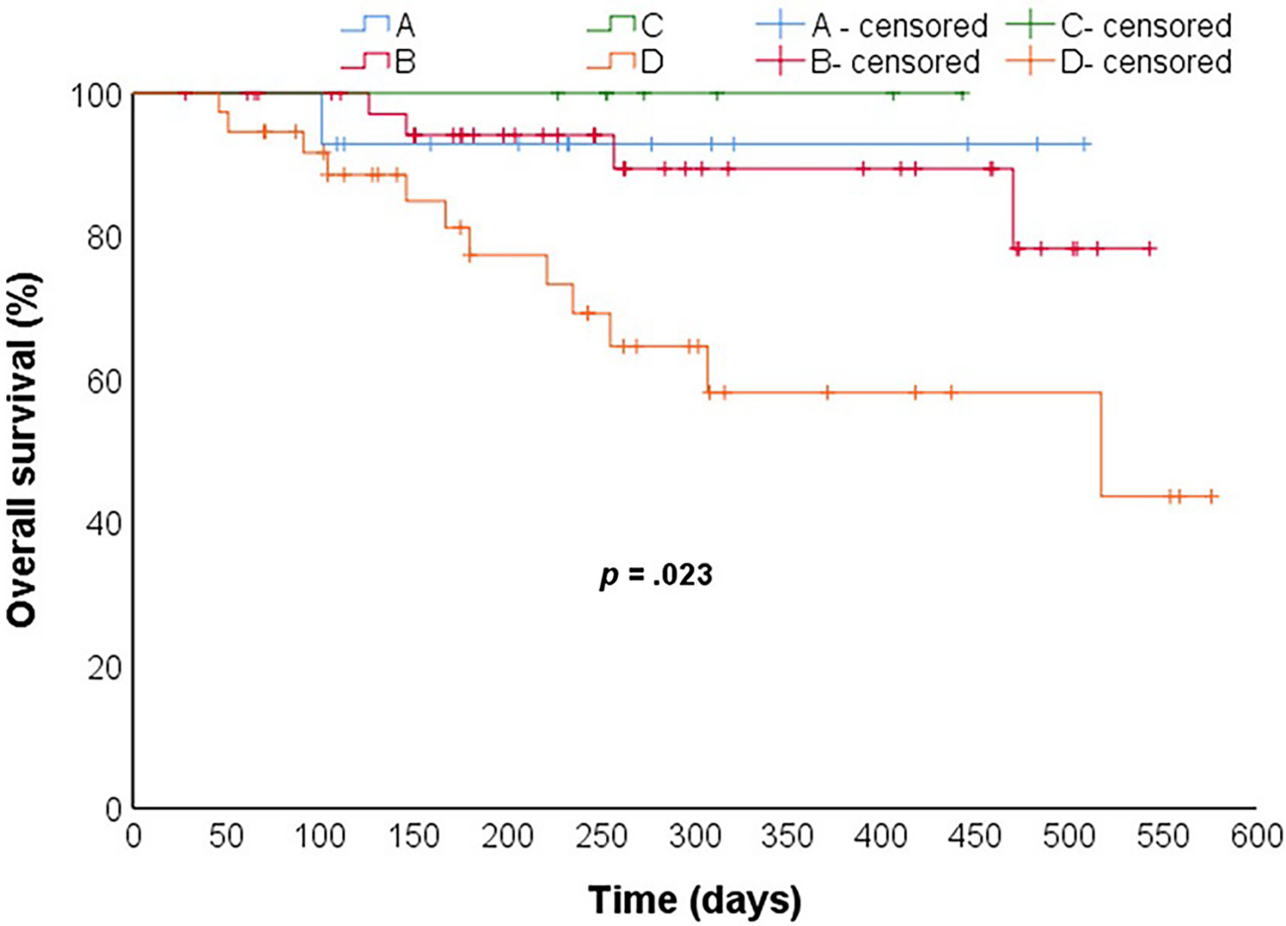
Kaplan–Meier estimates of the overall survival in the following group: (A) without cachexia and NLR ≤3; (B) without cachexia and NLR >3; (C) with cachexia and NLR ≤3; (D) with cachexia and NLR >3. A contrast with (a) B, *p* = .830; (b) A contrast with C, *p* = .480; (c) A contrast with D, *p* = .101; (d) B contrast with C, *p* = .432; (e) B contrast with D, *p* = .017; (f) C contrast with D, *p* = .082. NLR, neutrophil‐to‐lymphocyte ratio.

Previously published studies have shown that albumin and NLR levels are associated with the prognosis of cachexia.^18^, ^20^ We performed an exploratory analysis of the survival curves of four groups of patients divided based on the levels of albumin and NLR, respectively, (non‐cachexia and albumin ≤35 g/L; non‐cachexia and albumin >35 g/L; cachexia and albumin ≤35 g/L; cachexia and albumin >35 g/L) (non‐cachexia and NLR ≤3; non‐cachexia and NLR >3; cachexia and NLR ≤3; cachexia and NLR >3) (Figures 8 and 9).

The difference in the overall survival time distribution among the four groups was statistically significant (*p* = .002). Then, the survival time distribution of the four groups was compared in pairs. The significance level was corrected, and *p*‐values <.008 were considered to indicate statistically significant differences. The mean OS of patients presenting with cachexia and hypoproteinemia (81 days, 95% CI = 178–495) was significantly lower compared to those without cachexia and exhibiting normal protein levels (518 days, 95% CI = 484–552) (*p* < .001) (Figure 8).

The other four groups, which were divided according to the NLR values, were analyzed similarly. The difference in the overall survival time distribution among the four groups also was statistically significant (*p* = .023). The prognosis of the group with cachexia and high NLR was the worst. However, when any two groups were compared, the difference was not statistically significant (Figure 9).

### 3.5 Spearman correlation analysis

Spearman correlation analysis was conducted to test the associations between other factors, the data of which were collected, and BMI. Additionally, we evaluated the association between other factors and PMMA. We found that BMI, albumin, total protein, and HB were all positively correlated with PMMA (*r* = .302, *p* = .002; *r* = .250, *p* = .013; *r* = .342, *p* = .001; and *r* = .442, *p* < .001). We also found that HB and total protein were positively correlated with BMI (*r* = .316, *p* = .002 and *r* = .335, *p* = .001). The differences in the above results were statistically significant. Albumin and BMI were positively correlated but the differences were not statistically significant.

### 3.6 Effects of different factors on OS in immunotherapy

To evaluate the influence of other factors on the immunotherapy outcomes, we used Kaplan–Meier and log‐rank methods for survival analysis. Among the 98 patients who received immunotherapy, the patients with a baseline albumin >35 g/L (mean OS: 389 days, 95% CI: 287–490 days) had significantly better OS than patients with a baseline albumin ≤35 g/L (mean OS: 495 days, 95% CI: 458–532 days) (*p* = .001). Additionally, the patients with high‐albumin showed longer PFS than those with low (*p* = .018) (Figure S2). Next, we analyzed the relationships among PS, NLR, metastatic organs, and survival. The results are presented in the figures below (Figure S3 and Figure S4). The mean PFS was significantly shorter in the group with more metastatic organs (*p* = .025). However, we did not detect an association between more metastasis organs and longer OS (*p* = .682) (Figure S5).

## 4 DISCUSSION

In this retrospective study, we employed body weight in conjunction with PMMA to evaluate cachexia and examined its impact on clinical outcomes among 98 patients diagnosed with digestive system malignancies undergoing immunotherapy. Up to now, no study has investigated weight loss combined with PMMA changes to evaluate the occurrence of cachexia in patients with digestive system tumors. In this study, the incidence of cachexia is 45%. Previous studies have suggested that the incidence of cancer cachexia is about 23.8%–75%,^21^ with a wide range of prevalence. This large range of variability is influenced by cancer‐related (type, stage, and treatment), demographic (age) and diagnostic criteria. By using a combination of criteria based on BMI and percentage weight loss over time, Pressoir et al. found an overall prevalence of cachexia of 49.5% for upper digestive tumors.^22^ Rounis et al. used body weight and skeletal muscle index as assessment criteria. The prevalence of cachexia in non‐small cell lung cancer was 51.8%.^23^ Jo et al. diagnosed cachexia based on weight changes. In their study, the prevalence of non‐small cell lung cancer cachexia was 35.3%.^24^ A study on the prevalence of cachexia in older patients with colorectal cancer suggested that the incidence of cachexia in older patients with colorectal cancer was 36%.^25^ In most studies, the definition of cachexia only takes into account weight change. Although weight is also a manifestation of cachexia, weight is susceptible to progesterone and corticosteroids, which can relieve anorexia and increase weight but do not correct malignant cachexia.^26^ The number of patients with cachexia in these cohorts that account only for changes in body weight may be underestimated. Although skeletal muscle was mentioned in a few studies, they used skeletal muscle index to evaluate cachexia, which also differed from the evaluation method used in this study. The change rate of PMMA used in this study emphasizes the changes in skeletal muscle, which may better reflect the changes in the body composition of tumor patients during treatment. Numerous studies have demonstrated the reliability of assessing cachexia through the change of PMMA.^16^, ^17^ The study shows that AUC = 0.985 (≥0.90) is excellent. Therefore, the combination of PMMA and body weight measured during routine checkups has the potential to serve as an indicator for diagnosing cachexia.

Clinical indicators used to predict the effect of immunotherapy include PD‐L1 expression, tumor mutation load, and mismatched gene repair.^27^ However, the above indicators all have shortcomings. For example, different antibodies can detect different PD‐L1 positive reaction results. Radiotherapy, chemotherapy, and targeted therapy can also induce changes in PD‐L1 expression.^28^ Studies by Nishioka et al. have shown that the rate of change in PMMA can provide predictive information for immunotherapy.^29^ Our study shows that the presence of cachexia was significantly associated with poor OS in malignant tumors of the digestive system patients who received immunotherapy, not with PFS or the response to immunotherapy. Our conclusions are different from the results of previous studies. Werf et al. evaluated the clinical manifestations of cachexia in a study of patients with metastatic colorectal cancer. They found that patients with cachexia had shorter PFS than those without cachexia (*p* = .016). No other differences in outcomes were found between cachectic versus non‐cachectic patients.^30^ Jo et al., in a study on the treatment of advanced non‐small cell lung with pembrolizumab, evaluated cachexia and poor prognosis by weight change (PFS: 4.2 months vs. 7.1 months, *p* = .04, and OS: 10.0 months vs. 26.6 months, *p* = .03) were significantly associated with the efficacy of pembrolizumab.^24^ Another study on metastatic non‐small cell lung cancer demonstrated that cancer cachexia is associated with reduced response rates to PD‐1/PD‐L1 inhibitors and that it consists of an independent predictor for both inferior PFS and OS.^23^ These differences can be attributed to the small number of the 98 patients analyzed in our study, the short follow‐up time, and the different evaluation criteria for cachexia. In addition, cachexia has previously been reported to be more common in gastric cancer, up to 75%,^22^, ^31^ and generally lower in colorectal cancer.^21^ We then performed a subgroup analysis of these patients. In this study, the incidence of gastric cancer cachexia was 53% and colorectal cancer cachexia 37%. Cachexia negatively affected the prognosis of gastric cancer, but no significant difference was found in colorectal cancer. We acknowledge that the interpretation of these results is limited due to the small sample size and that the conclusions must be confirmed by subsequent studies on a larger scale.

Cancer cachexia syndrome (CCS) is a complex metabolic syndrome characterized by weight loss, alterations in the body composition, and a pathophysiologic background that is defined by a perpetually sustained inflammatory process.^32^ The NLR is a measure of systemic inflammation and has received increased attention in research on cancer and cachexia. Recently, accumulating evidence has suggested that cancer patients with cachexia have generally higher levels of NLR than those without cachexia, and high NLR adversely affects the OS of cancer patients with cachexia.^20^ Some studies also came to different conclusions. Richards et al. found that albumin and C‐reactive protein were related to cachexia. There was no relationship between cachexia and NLR.^33^ In this study, we demonstrated that the baseline albumin value was a significant positive prognostic biomarker for patients. However, no correlation was found between baseline NLR value and the prognosis of immunotherapy. Due to the small number of patients included, there were only 54 patients with cachexia, and the relationship between NLR and prognosis of patients with cachexia was not further analyzed. Interestingly, we divided patients into four groups based on NLR levels (non‐cachexia and NLR ≤3; non‐cachexia and NLR >3; cachexia and NLR ≤3; and cachexia and NLR >3). The difference in OS time distribution between different groups was statistically significant. Patients with cachexia combined with high NLR values had the shorter mean OS, suggesting a possible relationship between cachexia and NLR. However, when the four groups of patients were pairwise compared, there was no statistically significant difference between the groups. We think this may be related to the fact that many comparisons were made between the groups. In the future, we may collect more data from patients with cachexia to further analyze the relationship between NLR and cachexia.

CCS involves a broad spectrum of inflammatory processes. Other systemic inflammation‐related blood biomarkers and specific cytokines (such as C‐reactive protein, interleukins IL‐6, and IL‐8) are also commonly measured and used to predict clinical outcomes for patients with cancer.^19^, ^34^, ^35^, ^36^ However, our study did not examine and discuss the indicators mentioned above. The main reason is that our investigation is a retrospective analysis, and these indicators are not routine examination indicators. Most patients do not have these data recorded before starting immunotherapy.

In this study, we also confirmed the linear relationship between BMI, albumin, total protein, HB, and PMMA (r = .302, *p* = .002; *r* = .250, *p* = .013; *r* = .342, *p* = .001; and *r* = .442, *p* < .001), there was a linear relationship between total protein, HB, and BMI (r = .316, p = .002 and r = .335, *p* = .001). The above indicators could be used as an indicator of cachexia, but this is only our speculation, and further research is needed to verify it.

PS score can comprehensively evaluate the general condition of patients, and studies have shown that cachexia is related to the decrease in PS score.^18^ This study shows that cachexia can sensitively respond to OS in immunotherapy patients, but PS score is not associated with prognosis in immunotherapy. The main characteristic of cachexia is muscle loss. Because the disease consumes skeletal muscle, the appearance of cachexia may precede changes in PS.^18^ In addition, the limited duration of the follow‐up period led to no change in PS scores for some patients.

The main limitation of this study is that the sample size is relatively small. Second, the design was a single‐center retrospective, which led to lower validity of the collected clinical data and a limited number of factors included in the multivariate analysis. Although our study observed that cachexia is related to OS of immunotherapy, many factors affect immunotherapy, and the current study lacks systematic analysis of relevant indicators. For example, weight quantization score analysis of each influencing factor is carried out, and various scores are integrated to predict the effect of immunotherapy according to the highest score. Furthermore, other covariates and some unmeasured or measured confounders could have influenced the therapy, the mortality, and the results of our analyses. Therefore, our findings need to be confirmed in large prospective studies. It is also essential to explore whether early supportive interventions, such as targeted nutritional support, can positively impact treatment outcomes in patients receiving immunotherapy.

## 5 CONCLUSIONS

We demonstrated for the first time, using a CT scan to evaluate PMMA, that malignant tumors of the digestive system patients undergoing treatment with PD‐1/PD‐L1 inhibitors with cachexia at baseline show worse OS compared to patients without cachexia. The presence of cachexia was not associated with PFS or the response to immunotherapy. However, cachexia adversely affects the OS and PFS of gastric cancer patients, indicating cachexia is an independent prognostic indicator for patients with gastric cancer. PMMA measurement in CT images combined with body weight taken at routine checkups has the possibility of a reliable indicator for diagnosing cachexia. Therefore, it may be important to perform a baseline assessment of cachexia in malignant tumors of the digestive system patients before starting ICIs. However, we acknowledge that the sample size is too low to draw definitive conclusions. Research with a larger cohort is undoubtedly needed to confirm the above results. Future randomized controlled trials should be performed to confirm if early treatment of cachexia in the malignant tumor of the digestive system patients with PD‐1/PD‐L1 inhibitors is associated with improved overall survival.

## AUTHOR CONTRIBUTIONS

**Zhirui Tao:** Conceptualization; data curation; formal analysis; writing – original draft. **Zhiqin Chen:** Conceptualization; validation; writing – review and editing. **Yong Gao:** Conceptualization; validation; writing – review and editing. **Ming Quan:** Conceptualization; project administration; resources; writing – review and editing.

## FUNDING INFORMATION

This work was sponsored by National Natural Science Foundation of China [grant numbers 81972280, 81972290]; Natural Science Foundation of Shanghai Municipality [grant number 23ZR1452300]; Academic Leaders Training Program of Pudong Health Bureau of Shanghai [grant number PWRd2022‐02]; Research Grant for Health Science and Technology of Pudong Health Bureau of Shanghai [grant number PW2022E‐02].

## CONFLICT OF INTEREST

The authors have stated explicitly that there are no conflicts of interest in connection with this article.

## ETHICS STATEMENTS

This study was approved by the Ethics Committee of Shanghai East Hospital (Grant NO.2024‐035) and certify that the study was performed in accordance with the ethical standards as laid down in the 1964 Declaration of Helsinki and its later amendments or comparable ethical standards. Informed consent was obtained from all subjects and their legal guardians.

## Supporting information


**Figure S1.** Distribution of tumor types in the two groups.

**Figure S2.** Progression‐free survival (A) and overall survival (B) in the low‐albumin and high‐albumin groups; low‐albumin: albumin ≤35 g/L; high‐albumin: albumin >35 g/L.

**Figure S3.** Progression‐free survival (A) and overall survival (B) in the low‐PS and high‐PS groups; PS, performance status; low‐PS: PS 0–1; high‐PS: PS 2–3.

**Figure S4.** Progression‐free survival (A) and overall survival (B) in the low‐NLR and high‐NLR groups; NLR, neutrophil‐to‐lymphocyte ratio; low‐NLR: NLR ≤3; high‐NLR: NLR >3.

**Figure S5.** Progression‐free survival (A) and overall survival (B) in the No. of metastasis organs ≤3 and No. of metastasis organs >3 groups.

## Data Availability

The datasets analyzed during the current study are not publicly available due the privacy constraints, but are available from the corresponding author on reasonable request.
