# Supplementary figures and images for: Influence of cachexia on immunotherapy efficacy and prognosis for malignant tumors of the digestive system
Source: Cancer Rep (Hoboken). 2024 May 22;7(5):e2100. doi: 10.1002/cnr2.2100 (PMC11110103; doi:10.1002/cnr2.2100)

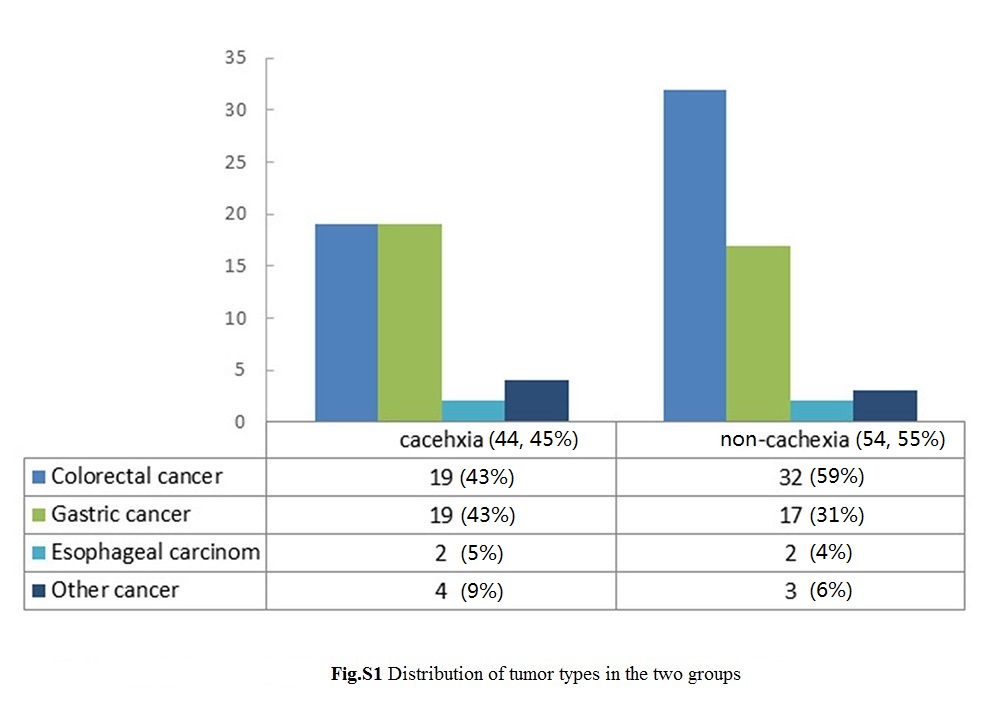

Supplement: Supplementary file 1 — Figure S1. Distribution of tumor types in the two groups. [file CNR2-7-e2100-s003.jpg]

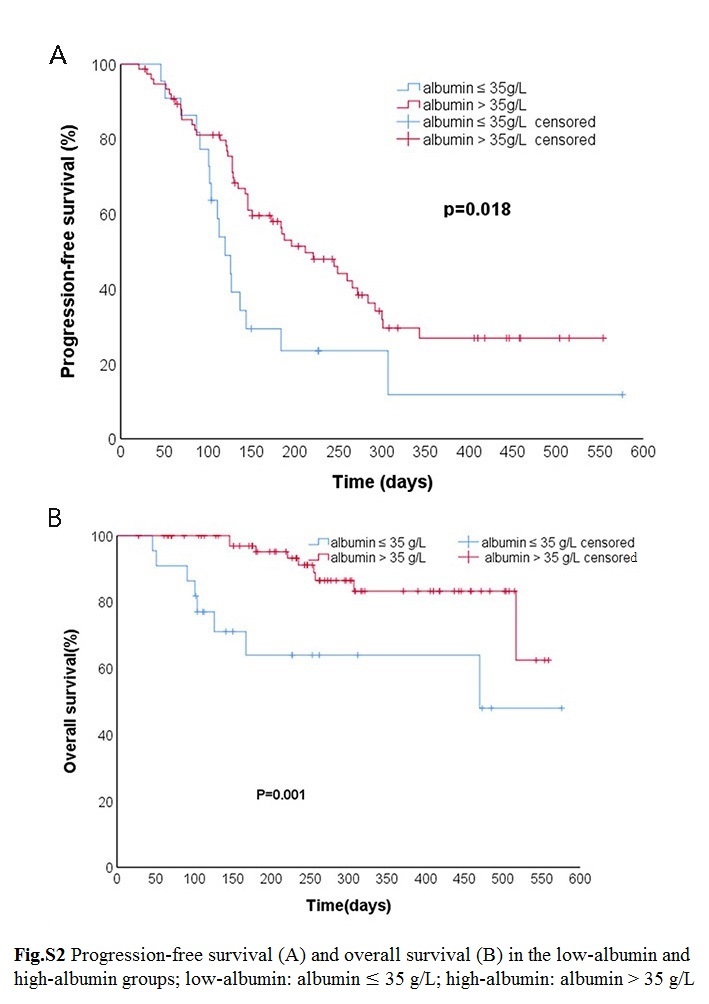

Supplement: Supplementary file 2 — Figure S2. Progression‐free survival (A) and overall survival (B) in the low‐albumin and high‐albumin groups; low‐albumin: albumin ≤35 g/L; high‐albumin: albumin >35 g/L. [file CNR2-7-e2100-s004.jpg]

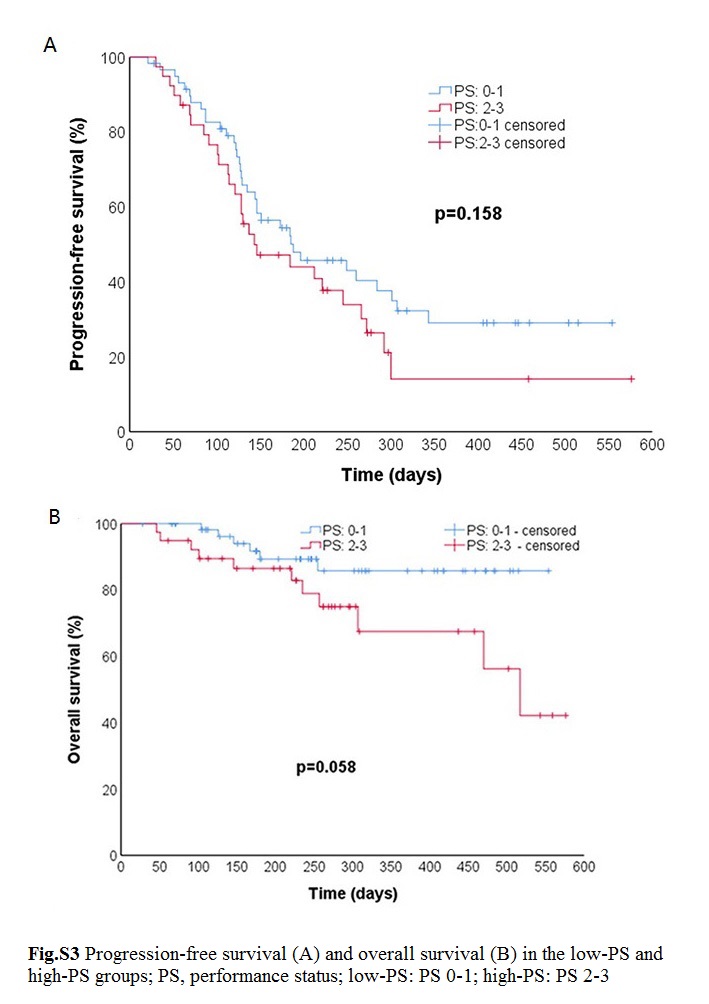

Supplement: Supplementary file 3 — Figure S3. Progression‐free survival (A) and overall survival (B) in the low‐PS and high‐PS groups; PS, performance status; low‐PS: PS 0–1; high‐PS: PS 2–3. [file CNR2-7-e2100-s001.jpg]

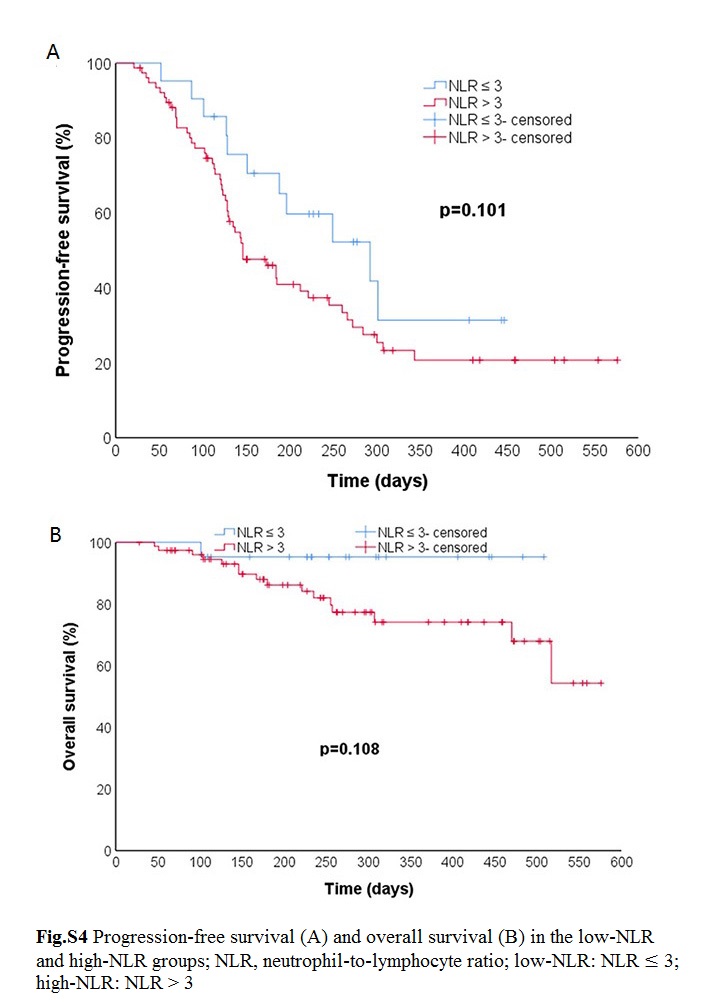

Supplement: Supplementary file 4 — Figure S4. Progression‐free survival (A) and overall survival (B) in the low‐NLR and high‐NLR groups; NLR, neutrophil‐to‐lymphocyte ratio; low‐NLR: NLR ≤3; high‐NLR: NLR >3. [file CNR2-7-e2100-s002.jpg]

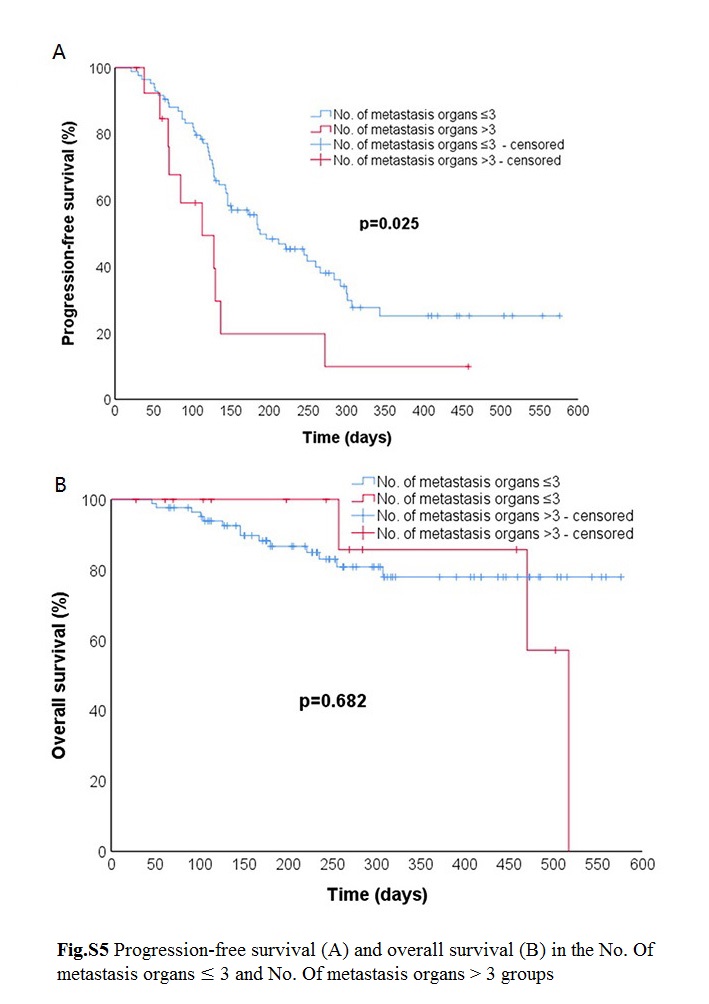

Supplement: Supplementary file 5 — Figure S5. Progression‐free survival (A) and overall survival (B) in the No. of metastasis organs ≤3 and No. of metastasis organs >3 groups. [file CNR2-7-e2100-s005.jpg]
